# A qualitative evidence synthesis using meta-ethnography to understand the experience of living with pelvic organ prolapse

**DOI:** 10.1007/s00192-020-04494-z

**Published:** 2020-09-01

**Authors:** Francine Toye, Jeannine Pearl, Katy Vincent, Karen Barker

**Affiliations:** 1 Nuffield Department of Orthopaedics, Rheumatology and Musculoskeletal Sciences (NDORMS), University of Oxford, Windmill Road, Oxford, OX3 7LD UK; 2 Suffolk, UK; 3 Nuffield Department of Women’s and Reproductive Health (NDWRH), University of Oxford, Oxford, UK; 4 Physiotherapy Research Unit, Oxford University Hospitals NHS Foundation Trust, Oxford, UK

**Keywords:** Meta-ethnography, Pelvic organ prolapse, Qualitative evidence synthesis, Qualitative research, Urogynaecology

## Abstract

**Introduction and hypothesis:**

Pelvic organ prolapse (POP) affects the lives of many people. We aimed to systematically search for, identify and synthesize qualitative research that explores what it is like to live with POP and make this knowledge available for healthcare improvement.

**Methods:**

We systematically searched Medline, PsychInfo, Embase and CINAHL, from inception to March 2020, for qualitative research exploring the experience of living with POP. We used *meta-ethnography* to synthesize findings. This is a conceptual approach to qualitative evidence synthesis. We used the recent guidelines for reporting meta-ethnography.

**Results:**

We screened 3103 titles and 255 abstracts and included 37 primary studies. These incorporated the experience of 777 women, (aged 18 to 95 years) from a range of countries. We organized 162 ideas into 27 conceptual categories and 10 themes. We developed a conceptual model that helps us to understand the experience of pelvic organ prolapse. This model indicates that (1) the physical losses of POP are intricately linked to loss of identity; (2) women conceptualized POP as part of womanhood, yet also its thief; (3) there is a vicious cycle of taboo, silence and misunderstanding about POP and its treatment; (4) this silence is exacerbated by a feeling that POP is not taken seriously in healthcare.

**Conclusions:**

This meta-ethnography helps us to understand the experience of living with a POP. Our model illustrates the complex process of healthcare decision making. Further studies to explore the complexity of decision making from the perspective of patient and health professional are timely.

## Introduction

Pelvic organ prolapse (POP) occurs when the muscles and tissues supporting the pelvic organs become weakened, causing one or more of the organs to bulge down out of position. Symptomatic POP has a prevalence of 3–6%, although this may be has high as 50% when based upon vaginal examination [1, 2]. In the UK, 96,286 surgical procedures for POP were performed between 2008 and 2017 [3]. This number is likely to increase with an ageing population. Public concern about the use of mesh for POP surgery, and the subsequent withdrawal of these procedures [3], has highlighted the importance of incorporating patient voices into healthcare policy and practice [4]. We aimed to systematically search for, identify and synthesize qualitative research exploring the experience of living with POP in order to understand this experience and incorporate this knowledge into healthcare improvement.

## Material and methods

### Ethics

Ethical permissions were not required for this study as it is an evidence synthesis of published studies.

Patient and Public Involvement: We identified a patient partner with experience of urogynaecology healthcare for POP to co-analyse the data and to work with us to develop a meaningful and relevant conceptual model. The National Institute of Health Research (UK) support the involvement of patient partners in research (https://www.invo.org.uk/) and we have found that patient partners make an important contribution to analysis in QES.

### Stage 1: Selecting meta-ethnography and getting started

This phase includes developing the rationale and aims of the study. To determine the need for a QES to explore the experience of living with POP, we first searched for any existing QES, using search terms designed for that purpose [5]. We found one QES which aimed to identify core treatment outcomes for POP [6]. Our innovation was to undertake a comprehensive search and conceptual synthesis of primary qualitative research using the methods of meta-ethnography to help us to understand what it like to live with POP. There are different methods for synthesizing the findings of qualitative research. Some reviewers focus on amalgamating and describing primary research findings, whereas others aim to abstract findings and develop conceptual understanding. We planned to conceptualize the experience of living with POP and to develop a line of argument synthesis to make ‘a whole into something more than the parts alone imply’ [7] (page 28). Meta-ethnography is a conceptual approach [7]. This approach has been used effectively to conceptualize the experience of urinary incontinence [8]. We used the recent guidelines for reporting meta-ethnography (eMERGe) [9]. These guidelines report recommendations, guidance and good practice for conducting the seven phases of a meta-ethnography.

### Stage 2: Deciding what is relevant

We included qualitative studies that explored the experience of POP. We used thesaurus and free text terms for qualitative research, combined with thesaurus and free text terms for POP. We limited our search to studies reported in English. Table 1 reports the elements of STARLITE recommended for qualitative research. STARLITE is an acronym which outlines the standards recommended for reporting systematic searches of qualitative research: Sampling strategy, Type of study, Approaches, Range of years, Limits, Inclusion and exclusions, Terms used and Electronic sources [10]. We began with Medline and then proceeded with PsychInfo, CINAHL and EMBASE to allow us to evaluate the added value of searching these databases. A single reviewer with > 20 years of qualitative research experience screened titles and abstracts for relevance [7]. Two reviewers appraised full texts, excluded ‘irrelevant’ or ‘fatally flawed’ studies and included studies that were at least ‘satisfactory’ [11].

**Table 1:** Reports the elements of STARLITE: Sampling strategy, Type of study, Approaches, Range of years, Limits, Inclusion and exclusions, Terms used, Electronic sources

Starlite category	Description
Sampling strategy	Comprehensive
Type of studies	Qualitative research, fully reported
Approaches	Electronic databases
Range of years	To April 2020
Limits	[Languages English]
Inclusions and exclusions	Pelvic organ prolapse Excluded: mixed samples where unable to decipher experience of POP from other experience
Qualitative methods - thesaurus terms	**MEDLINE:** exp. “FOCUS GROUPS”/ OR exp. “ANTHROPOLOGY, CULTURAL”/ OR exp. “QUALITATIVE RESEARCH”/ OR exp. “NURSING METHODOLOGY RESEARCH”/ OR exp. “INTERVIEWS AS TOPIC”/ **PSYCHINFO:** exp. “THEMATIC ANALYSIS”/ OR exp. “SEMI-STRUCTURED INTERVIEW”/ OR exp. “NARRATIVE ANALYSIS”/ OR exp. “INTERPRETATIVE PHENOMENOLOGICAL ANALYSIS”/ OR exp. “GROUNDED THEORY”/ OR exp. “FOCUS GROUP”/ OR exp. “QUALITATIVE METHODS”/ OR exp. PHENOMENOLOGY/ OR exp. ETHNOGRAPHY/ OR exp. “GROUP DISCUSSION”/ **CINAHL:** exp. “PHENOMENOLOGICAL RESEARCH”/ OR exp. “GROUNDED THEORY”/ OR exp. “ETHNONURSING RESEARCH”/ OR exp. “ETHNOLOGICAL RESEARCH”/ OR exp. “ETHNOGRAPHIC RESEARCH”/ OR exp. “ACTION RESEARCH”/ OR exp. “NATURALISTIC INQUIRY”/ OR exp. “QUALITATIVE STUDIES”/ OR exp. “ANTHROPOLOGY, CULTURAL”/ OR exp. “FOCUS GROUPS”/ OR exp. “DISCOURSE ANALYSIS”/ OR exp. “CONSTANT COMPARATIVE METHOD”/ OR exp. “PURPOSIVE SAMPLE”/ **EMBASE:** exp. HERMENEUTICS/ OR exp. “QUALITATIVE RESEARCH”/ OR exp. PHENOMENOLOGY/ OR exp. “PERSONAL EXPERIENCE”/
Qualitative methods – free text	Qualitative ADJ5 (theor* OR study OR studies OR research OR analys*)).ti,ab OR (ethnog*).ti,ab OR (phenomenolog*).ti,ab OR (hermeneutic* OR heidegger* OR husserl* OR colaizzi* OR giorgi* OR glaser OR strauss OR (van AND kaam*) OR (van AND manen) OR ricoeur OR spiegelberg* OR merleau).ti,ab OR (constant ADJ3 compar*).ti,ab OR (grounded ADJ3 (theor* OR study OR studies OR research OR analys*)).ti,ab OR (narrative ADJ3 analys*).ti,ab OR (discourse ADJ3 analys*).ti,ab OR (conversation ADJ3 analys*).ti,ab OR ((lived OR life) ADJ3 experience*).ti,ab OR ((theoretical OR purposive) ADJ3 sampl*).ti,ab OR (field ADJ note*) OR (field ADJ record*) OR fieldnote*).ti,ab OR (participant* ADJ3 observ*).ti,ab OR (action ADJ research).ti,ab OR (digital ADJ record) OR audiorecord*).ti,ab OR (co AND operative) AND inquir* OR co-operative AND inquir*).ti,ab OR ((semi-structured OR semistructured OR unstructured OR structured) ADJ3 interview*).ti,ab OR (feminis*).ti,ab OR (humanistic OR existential OR experiential).ti,ab OR (social AND construct*).ti,ab OR (poststructural* OR post structural* OR post-structural*).ti,ab OR (postmodern* OR post modern* OR post-modern*).ti,ab OR (‘appreciative inquiry’).ti,ab OR (‘interpretative phenomenological analysis’).ti,ab OR (face ADJ3 interview*).ti,ab OR ((depth OR in-depth) ADJ3 interview*).ti,ab OR (abductive ADJ analys*).ti,ab)
Condition terms	exp “WOMEN’S HEALTH SERVICES”/ OR exp. GYNAECOLOGY/ OR exp. “REPRODUCTIVE MEDICINE”/ OR exp. UROLOGY/)” AND exp. “PELVIC ORGAN PROLAPSE”/” AND exp. “URINARY INCONTINENCE”/” AND (prolapse).ti,ab
Electronic sources	Medline, PsychInfo, Cinahl, Embase

### Stage 3: Reading included studies

Once we had agreed which studies to include, we uploaded the manuscript onto Nvivo 11 software to allow us to keep track of data and link it to developing ideas. One reviewer read all studies in alphabetical order, by author, to identify concepts. Similarly, a second reviewer read the papers to identify, compare and discuss any differences. The aim of this was to add to concepts rather than agree about them.

### Stages 4 and 5: Determining how studies are related and translating studies

We included all concepts identified from different contexts and research designs. We extracted contextual information to allow us to determine how studies were related to each other. Two reviewers ‘translated’ the concepts between studies by comparing them with each other, distilling their essence and sorting them into *conceptual categories*. We identified any *disconfirming cases* [9] that did not support our interpretations. One reviewer translated each conceptual category into accessible first-person English to distil its essential meaning, and this was checked by our patient partner. We repeated the same process of constant comparison with our conceptual categories to develop final further abstracted themes. We used the four domains of the GRADE-CERQual framework [12] to encourage reflection: (1) methodological limitation, (2) relevance, (3) *adequacy* of data (‘richness and quantity of data’), and (4) *coherence* (‘consistency across studies’). It is currently the only framework of its kind designed to provide guidance for assessing how much confidence to place in findings from QES.

### Stages 6 and 7: Synthesizing translations and expressing the synthesis

We organized the themes into a conceptual model. This is done through a process of comparison, thinking and discussion: multiple draft versions of a model are made before reaching a final agreement on a model that synthesizes ideas into a line of argument. In view of the social distancing measures in place in response to COVID-19, two reviewers met on three occasions via remote meetings with video links to discuss and develop the model. We had successfully used this method when working with international patient partners on previous studies.

## Results

**Fig. 1:**
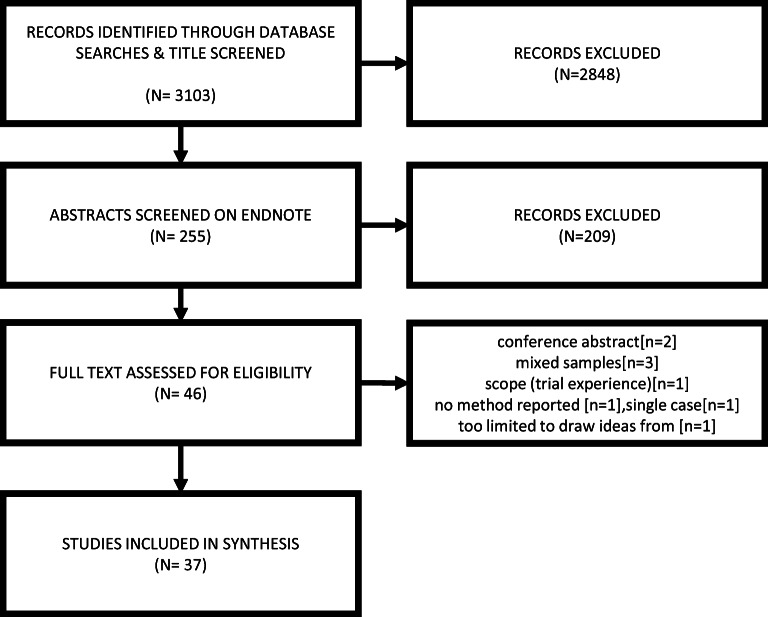
Search results, showing number of records identified, excluded and included

A summary of our search is shown in Fig. 1. We screened 3103 titles, 255 abstracts and 46 full texts: we excluded 9 full texts and included data from 37 studies (32 unique samples). The majority of studies (32 out of 37) were identified from MEDLINE [13–44]. Five further studies were identified from the remaining databases combined [45–49]. Table 2 shows the author, year of publication, number and age of participants, country of origin, condition, data collection and analysis methods, and aims for each study. Studies incorporated the experience of 777 women, ranging in age from 18 to 95 years, from a range of countries: USA (12), Ethiopia (4), Nepal (4), UK (4), New Zealand (4), Sweden (2), USA/Mexico border (2) Canada (1), Iran (1), Mexico (1), The Netherlands (1) and South Africa (1).

**Table 2:** Reports the author, year of publication, number and age of participants, country of origin, condition, data collection and analysis methods, and aims for each study

Author(S), year	Participants (same sample)	Age range (mean)	Country	Condition	Data collection	Analysis	Study aim
Abhyankar et al. 2019[13]	22	NK	UK	POP	Interviews/focus groups	Thematic analysis	Women’s experiences of seeking diagnosis and treatment for POP
Alas et al. 2016[41]	58 ^a^	(57) English (64) Spanish	USA	POP	Focus groups	Grounded theory	Perceptions of Spanish- and English-speaking women with POP
Baskayne et al. 2014[14]	28	32–86	UK	POP surgery	Interviews	Thematic analysis	Expectations of prolapse surgery and reasons why expectations were met or not met
Basu and Duckett 2009[15]	17	33–76	UK	POP/UI	Interviews	Thematic analysis	Why women with recurrent urogynaecology symptoms do not seek treatment
Basu, Wise and Duckett 2011[16]	16	48–70	UK	POP/UI	Iinterviews	Thematic analysis	Treatment decision-making process for stress urinary incontinence (SUI) and prolapse
Blystad et al. 2018[17]	5	NK	Ethiopia	POP	Interviews	Systematic text condensation	Reasons for under-reporting of POP in the Dabat Incontinence and Prolapse Study
Bonetti, Erpelding and Pathak 2004[45]	24	NK	Nepal	POP	Focus groups	Ethnography	Experience of prolapse and its perceived causes and consequences
Brown 2019[42]	7	43–69	NZ	POP/UI surgery	Interviews	Hermeneutic phenomenology	Women’s lived experience of pelvic surgical mesh complications
Chalise, Steenkamp and Chalise 2016[18]	21	25–60	Nepal	POP	Interviews	Thematic analysis	Factors affecting women seeking surgical treatment for POP at mobile surgical camps
Dunivan et al. 2014[19]	(58) ^a^	33–90	USA	POP	Focus groups	Grounded theory	English- and Spanish-speaking women’s experience with POP
Ghetti et al. 2015[20]	44	(60)	USA	POP	Interviews/focus groups	Thematic analysis	The emotional burden experienced by women seeking treatment for POP
Gjerde et al. 2017[23]	24 ^b^	24–65	Ethiopia	POP surgery	Ethnography/ focus groups/interview	Systematic text condensation	How women in a low-income setting explain, experience and handle consequences of POP
Gjerde et al. 2018[21]	(24) ^b^	24–65	Ethiopia	POP surgery	Ethnography/ focus groups/interview	Case stories	Recovery after free surgical treatment for POP in a resource-constrained setting
Gjerde et al. 2018B[22]	(24) ^b^	24–65	Ethiopia	POP surgery	Observation/interviews	Thematic analysis	Experiences of healthcare of women with severe POP in impoverished settings
Hadizadeh-Talasaz et al. 2019[46]	20	28–65	Iran	POP	Interviews	Content analysis	The sexual experience of women with POP
Hyland, Hay-Smith and Treharne 2014[24]	5	46–60	NZ	POP	Interview	Interpretative phenomenology	Post-supervised treatment adherence to pelvic floor muscle training for POP
Jackson et al. 2017[25]	24	24–95	USA/Mexican border	POP/UI	Focus groups	Grounded theory	Perceptions of POP/incontinence in Spanish-speaking Latinas on the US/Mexico border
Kiyosaki et al. 2012[26]	20	31–87	USA	POP/UI	Interview	Grounded theory	Effect of visit with a specialist on understanding of pelvic floor disorders
Low and Tumbarello 2012[27]	14	33–81	USA	POP	Interview	Framework of knowledge	How women comprehend, conceptualize and communicate their experiences with POP
Lowder et al. 2011[28]	25	(67)	USA	POP	Focus groups	Grounded theory	Perceptions of prolapse-specific body image in women with symptomatic prolapse
Maldonado et al. 2020[43]	29	40–79	USA/Mexican border	POP	Focus groups	Grounded theory	Pessary use in Spanish-speaking women along the US-Mexico border
Mirskaya, Lindgren and Carlsson 2019[47]	33	NK	Sweden	POP	Online forum	Thematic analysis	Fertile women’s experiences of symptomatic pelvic organ after vaginal birth
Muller 2010[48]	33	(64)	NZ	POP	Phone interviews	Thematic analysis	Impact of POP, experience of healthcare and treatment priorities
O’Dell and Jacelon 2005[29]	6	61–85	USA	POP surgery	Interviews	Phenomenology	The nature and range of the experience of vaginal closure surgery
Pakbaz et al. 2010[30]	14	42–79	Sweden	POP	Interviews	Thematic	Experiences of living with POP and its impact on daily life, prior to surgical intervention
Radl, Rajwar and Aro 2012[44]	71	NK	Nepal	POP	Focus groups	Grounded theory	The status of uterine prolapse prevention in Eastern Nepal
Roets 2007[49]	19	48–77	South Africa	POP	Interviews	Phenomenology	The experience of women with POP
Roos et al. 2014[31]	37 ^c^	31–64	The Netherlands	POP/UI surgery	Interviews	Data matrices	The impact of POP and/or UI on female sexual dysfunction
Roos et al. 2013[32]	(37) ^c^	31–64	The Netherlands	POP/UI surgery	Interviews	Data matrices	Condition-specific sexual function questionnaire after pelvic floor surgery
Sevilla et al. 2013[34]	27	41–71	USA	POP/UI	Interviews	Grounded theory	Impact of an initial specialist visit on Spanish-speaking women with pelvic floor disorders
Sevilla et al. 2013b[33]	16	47–85	USA	POP Pessary	Interviews	Grounded theory	Experiences of Spanish-speaking women who choose a pessary
Shrestha et al. 2014[35]	16	23–82	Nepal	POP	Interviews	Deductive analysis	Experiences of POP and healthcare-seeking practices
Smith-Oka 2014[36]	53	18–73	Mexico	POP	Observation/interviews	Ethnography	Experience of POP: focus on reproduction, motherhood and healthcare
Storey et al. 2009[37]	11	60+	Canada	POP/UI pessary	Interviews	Narrative inquiry	Experiences of women using pessaries for the treatment of incontinence or POP
Sung et al. 2014[38]	25	40–84	USA	POP surgery	Focus groups	Content analysis	To develop a conceptual framework for the most important outcomes for POP
Wieslander et al. 2015[39]	(58) ^a^	33–90 English 46–77 Spanish	USA	POP	Focus groups	Grounded theory	Experience and understanding of POP in Spanish- and English-speaking women
Zielinski et al. 2009[40]	13	33–81	USA	POP	Phone interviews	Content analysis	Body image questionnaire in women with pelvic organ prolapse

^a, b, c^ report the same sample

**Fig. 2:**
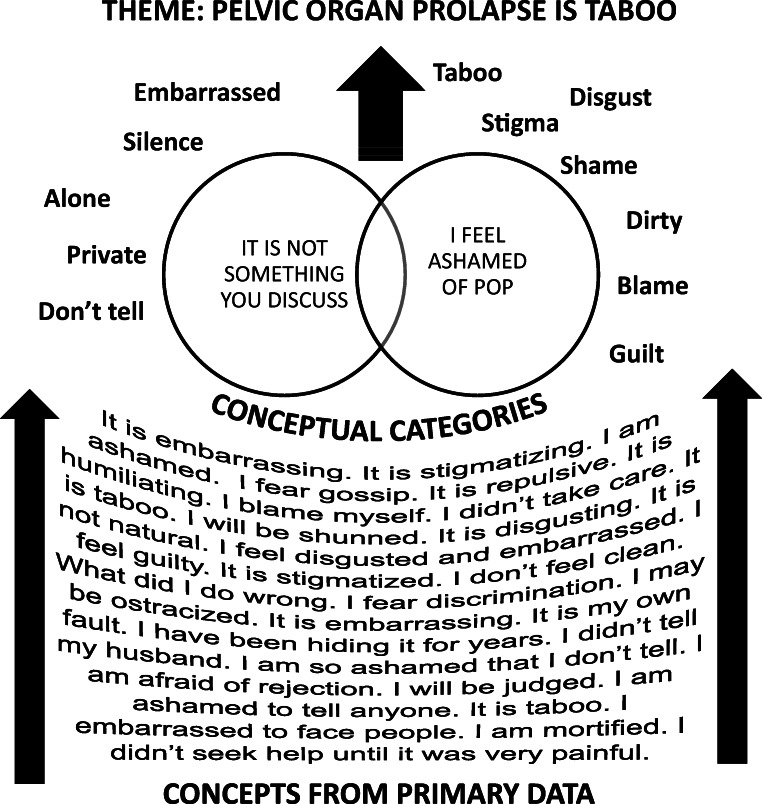
POP is taboo: An example of theme development. The aim of the analysis is to distil the essence of the data into discrete and useful ideas. At the bottom of the diagram, analysis starts with a body of data extracted from the primary studies (concepts). Through a process of careful reading and constant comparison, we categorize concepts into groups (conceptual categories). In this example, category A (POP is not something you discuss) arises from concepts such as: do not tell, keep silent, alone; category B (I feel ashamed of POP) arises from concepts such as stigma, taboo, shame. The boundaries between conceptual categories are not always solid, and concepts can fall into more than one category. It is at this stage that collaboration with others, in particular patient partners, can add to the rigour of a study. The next stage is to further abstract the categories into overarching themes. In this example, we felt that the overriding theme stigma and silence encompassed the two categories

We organized 162 ideas from the primary studies into 27 conceptual categories and 10 themes: my body is broken; the life of a woman can take its toll; I am broken; it has taken the woman out of me; my world is shrinking; pelvic organ prolapse is taboo; what on earth is going on down there; powerless in healthcare; which treatment should I choose; it was a relief to tell someone about it. Table 3 shows the studies supporting each theme. We report each theme with its underlying conceptual categories, translated into accessible first-person English. Figure 2 gives an example of the phases of analysis in developing a theme. We also identify any disconfirming cases that did not support our interpretations [9]. We do not include first person narratives.

**Table 3:** Reports the studies supporting each theme

**Author (s), year**	My body if broken	The life of a woman can take its toll	I am broken	It has taken the woman out of me	My world is shrinking	Pelvic organ prolapse is taboo	What on earth is going on down there?	Powerless in healthcare	What treatment should I choose	It was a relief to tell someone
Number of studies	**21**	**13**	**19**	**20**	**12**	**27**	**16**	**14**	**17**	**14**
Abhyankar et al. 2019[13]	x	x	x			x	x	x	x	x
Alas et al. 2016[41]^a^						x	x	x		x
Baskayne et al. 2014[14]	x	x							x	
*Basu and Duckett 2009[15]			x						x	x
*Basu, Wise and Duckett 2011[16]	x	x	x			x	x		x	
Blystad et al. 2018[17]				x		x				
Bonetti, Erpelding and Pathak 2004[45]	x	x		x	x	x				
Brown 2019[42]	x	x	x	x	x	x		x	x	x
Chalise, Steenkamp and Chalise 2016[18]	x					x	x			
Dunivan et al. 2014[19] ^a^				x		x	x	x		x
Ghetti et al. 2015[20]	x		x	x		x			x	x
Gjerde et al. 2017[23]^b^	x	x		x	x	x	x			x
Gjerde et al. 2018[21] ^b^	x	x		x	x	x			x	x
Gjerde et al. 2018B[22] ^b^	x	x	x	x		x	x		x	x
Hadizadeh-Talasaz et al. 2019[46]	x	x		x		x				
Hyland, Hay-Smith and Treharne 2014[24]									x	
*Jackson et al. 2017[25]							x	x		x
*Kiyosaki et al. 2012[26]		x					x	x	x	
Low and Tumbarello 2012[27]							x	x		
Lowder et al. 2011[28]	x		x	x	x	x				x
Maldonado et al. 2020[43]						x			x	
Mirskaya, Lindgren and Carlsson 2019[47]	x		x	x	x	x	x	x	x	
Muller 2010[48]								x	x	
O’Dell and Jacelon 2005[29]			x	x	x	x	x	x	x	
Pakbaz et al. 2010[30]	x	x	x	x		x	x	x		x
Radl, Rajwar and Aro 2012[44]					x	x				
Roets 2007[49]	x	x	x	x		x	x			
*Roos et al. 2014[31]^c^	x			x		x				
*Roos et al. 2013[32]^c^			x	x		x				
*Sevilla et al. 2013a[34]	x		x	x			x	x	x	
Sevilla et al. 2013b[33]			x						x	
Shrestha et al. 2014[35]	x	x	x	x	x	x		x		x
Smith-Oka 2014[36]	x	x			x	x				x
*Storey et al. 2009[37]			x		x	x			x	
Sung et al. 2014[38]	x	x	x	x	x	x				
Wieslander et al. 2015[39]^a^	x					x	x	x		
Zielinski et al. 2009[40]				x						

*Sample includes the experience of urinary incontinence

### My body is broken

This theme describes the physical losses of POP: my bladder and bowel are in a mess; it feels like I have a ball in my vagina; the pain and discomfort are unrelenting; my body is falling apart.

My bladder and bowel are a mess.

It can be difficult to empty my bladder or bowel because the prolapse gets in the way. It feels like I am giving birth when I go to the toilet. I have to use my fingers to splint my pelvic organs when I strain. I don’t eat because I am worried about getting constipated, and I take regular laxatives.

It’s like having a ball in your vagina.

It feels like I have a ball in my vagina. It is a bulge. I feel that something is going to fall out of me. It is an awful feeling that I am *open*. It pops out when I strain.

Unrelenting pain and discomfort.

I have had unrelenting discomfort and pain for years. I cannot bear it. I feel pressure, heaviness, fullness. My back and tummy hurt. I get painful spasms. My prolapse causes friction, and my vagina and rectum get itchy and sore. I get infections, discharge, ulceration and bleeding.

My body is falling apart.

I cannot even do ordinary things: I cannot sit, I cannot walk, I cannot run, I cannot exercise, I cannot lift things, I cannot work. I have to avoid or limit things. My body is falling apart. I live in a body that I cannot rely on. My body has lost its integrity. My body is no longer whole. It is irreparably damaged.

### The life of a women can take its toll

This theme describes prolapse as an inevitable consequence of women’s labour, both work and childbirth. There was a sense this burden is rooted in their gendered roles and that women must get used to it.

The labour of childbirth.

I had a difficult pregnancy and childbirth, I had lots of children, I didn’t rest after I gave birth and didn’t get any help. The body loses strength with every child. It is part of being a woman.

Enduring women's work.

The work that women have to endure takes its toll. I have done lots of heavy work. We have no time to rest. I am responsible for the household chores, childcare and cooking: I do heavy work. Hard work overwhelms us, and we don’t always get help.

We learn to live with it.

I have to be pragmatic. I got used to it. I have had time to accept it and adjust. I can get treatment, but I have become resigned to it. I have had to accept and focus on what remains.

We found one disconfirming case in rural Mexico [36], which not only questioned this inevitability, but also gave a sense that although the body loses strength with every child, that children also brought strength and support [36].

### I am broken

This theme describes loss of self-identity: I can no longer be fully present in what I do, I feel crushed, and I am no longer me.

I am not fully present in what I do.

My body, its sensations and limitations are at the forefront of every waking moment. I am distracted by it. I am always focusing on it. I constantly worry. It is always on my mind.

I am being crushed.

I am mentally broken. I cannot enjoy myself. I am sad. I am stressed. I am angry. I am frustrated. I am desperate. It has turned my life into a living hell. I cannot take it anymore. It feels like a life sentence.

I am no longer me.

I am having an identity crisis. I compare myself to what I was before. I cannot do the things that I used to love doing. I can only do what is necessary. My body is not me. I was strong. I was independent. I worked hard and was proud. I was enterprising and active. I never ask for help. I did not complain. I long for my old self.

### It has taken the woman out of me

This theme describes lost femininity and sexuality: I see no beauty in me, I have no sexual desire, and I worry that my partner will leave.

I see no beauty in me.

I no longer like my naked body and I don’t want my husband to see it. My vagina is not normal: it is big, loose and ugly. I am no longer attractive or desirable. I no longer feel like a woman. I see no beauty in myself. I feel old. I grieve my sexuality. I have lost my femininity.

I have no sexual desire.

I have lost the desire for sex and avoid it. I am tired out. Sex is painful. I have no sensation. I don’t initiate sex. I grin and bear it. I hurry through it. I worry about what is happening *down there*: I might damage myself; it might hurt; my partner might see it or feel it; I might wet the bed or make it dirty. I can’t give myself to the moment. I cannot be spontaneous: I have to wash and wee first; I have to push the prolapse inside: I can only be in certain positions. It is a real turn off.

I am worried that my partner will leave.

I worry about my partners sexual needs. I am worried that my partner will abandon me. I fake orgasm. I have even thought about letting my partner have sex with someone else. My partner no longer respects me and has become violent, humiliates and insults me. Not all are like that.

### My world is shrinking

This theme describes the loss of connection to the world: I feel like a social outcast and I am living in a gap between what was and what could have been.

I feel like a social outcast.

I have lost connection with others. I have become socially isolated. I no longer invite people to the house or get out. I rely on my family to do things, and they can no longer depend on me. My in-laws humiliate me because I cannot do the things that wives and mums should do.

Living in a gap between past and future.

I am stranded in the present. I am living in a gap between what was and what could have been. What once seemed like a world of opportunity now feels contracted. My world is shrinking. I have lost my sense of wholeness and being-in-the-world. I worry about it getting worse. I have lost hope for the future. I am lost.

### Pelvic organ prolapse is taboo

This describes the sense of stigma and silence that dominate the experience of living with POP: I feel ashamed and it is not something that you can discuss.

I feel ashamed.

I am ashamed to tell anyone. It is embarrassing and stigmatising. It is taboo. I fear gossip and discrimination. It is humiliating. People will judge and shun me. I cannot face the shame of my family knowing. I feel disgusting. I cannot control my own body. I feel unclean. It is so unpleasant. I blame myself. I must have done something wrong. I kept lifting heavy things. I did not do my exercises.

It is not something you discuss.

Prolapse is hard to talk about it. There are no easy words. I don’t even know what to call it. It is too intimate to discuss. I suffer in silence. I have been hiding it for years. I don’t tell my family or friends, or even other women. I only told my husband when I could no longer hide it. I didn’t even tell my doctor. I feel alone.

### What on earth is going on down there?

This theme describes a lack of understanding exacerbated by the silence surrounding POP: I do not understand what it is and I am frightened. However, I do not want to ask anyone about it.

I don’t understand what a prolapse is.

I don’t really know what is going on down there: I don’t understand the anatomy; I don’t understand what a prolapse is. You hear about incontinence but never about prolapse. If I hadn’t looked, I wouldn’t have found out about it. The information on the internet was overwhelming. I don’t want to ask questions.

I was frightened that it was cancer.

I was so scared. I didn’t know what was happening to me. I was worried that it was cancer or an infection. I am worried that I might develop cancer if I don’t seek treatment soon.

We found one disconfirming case of women living on the USA/Mexico border [25], challenging this lack of understanding, although the study highlights women’s misconceptions about the cause of POP.

### Powerless in healthcare

This theme describes a feeling of being powerless in healthcare: the health professional undermines my experience: they do not take it seriously and they do not know what they are doing.

The health professional undermines my experience

I thought that I had a problem, but the health professional said that I didn’t. I started to doubt myself. I just accepted what they said. They don’t discuss it with you. I was left with more questions than answers. I feel like a nuisance. They did not tell me that prolapse could get worse or discuss the importance of exercise. I feel misled.

The health professionals don’t take it seriously.

The health professional dismissed my prolapse as ‘just bagginess’. They don’t take me seriously. They trivialize POP. They make you think that you are making a big fuss. They say it doesn’t look too bad and that it is very common. There is no sense of urgency.

The health professionals don’t know what they are doing.

The health professional does not seem to know about prolapse or how to treat it. You get different diagnoses. I am shocked about how little they know. They all have different ideas about it. I don’t even know if they are choosing the right treatment.

### Which treatment should I choose?

This theme describes the complexity of making treatment decisions for prolapse: It is difficult to get into the habit of exercising; you have to learn to live with a pessary; surgery might not solve the problem.

It is difficult to get into the habit of exercising.

I find it difficult to do pelvic floor muscle exercises regularly and accurately. I forget. I am not confident how to do it properly. I don’t know if I am doing it right. You have to get into an exercise routine. Sometimes other things take precedence: I put my family needs first.

You have to learn to live with a pessary.

I hadn’t heard of a pessary and didn’t know what it was. I was worried that my body might reject it, or that it might get stuck. I did not feel confident about removing and inserting it. I was not comfortable touching myself. It takes time to get used to the idea. It is not a cure. It can be uncomfortable, or cause bleeding or discharge. However, it gave me freedom to choose when to use it and to do things that I could not do before. It is also less risky than surgery.

Surgery might not solve the problem.

I just wanted to have surgery so that it was gone for good: a pessary or exercises will not *cure* me. However, I have to balance the risks and benefits: Surgery might be too risky for me; it might not get rid of my symptoms; I might still be incontinent; I might need another operation; it might come back; there might be complications; I had already had unsuccessful surgery; do I really want plastic bits down there? If surgery fails, the impact would be potentially devastating.

### It was a relief to tell someone about it

This theme describes the relief that can follow if you break the silence of POP: I let it get so bad before I asked for help and it was a relief to know that I was not alone.

I let it get so bad before I asked for help.

I should have done something sooner. I let it get so bad. However, I had to weigh up lots of things before I decided to ask for help: It is so embarrassing; it seems trivial compared to other things; I didn’t have the money for travel or treatment; my life is too busy.

It was a relief to know that I was not alone.

It is a relief to tell people. I wish I had spoken sooner. It was so good to talk to someone else with POP. We shared our experience. I realised that I was not on my own, and I felt better. I am much more open now, and people are sympathetic. We don’t have to hide. I have become an advocate for other women with POP and that makes me feel good.

### Conceptual model

**Fig. 3:**
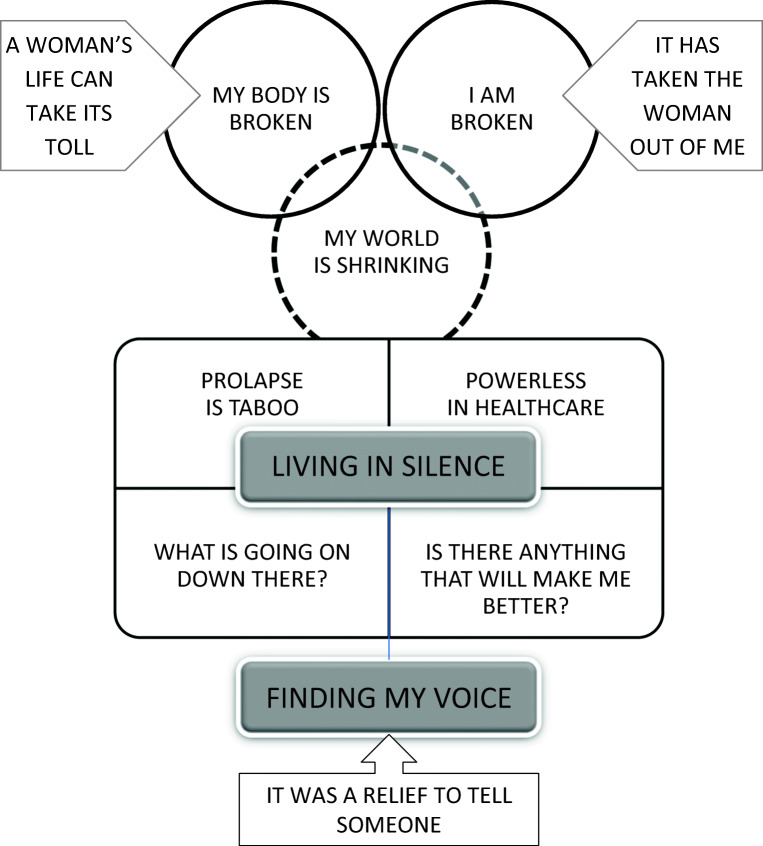
Conceptual model, illustrating the line of argument which is described in the manuscript

We developed a conceptual model that can help us to understand the experience of living with POP (Fig. 3). The model starts with physical losses (*my body is broken*), closely linked to loss of identity (*I am broken*): I can no longer do the things that I normally do, and I am losing my identity. Time and social space are shrinking, and I am living alone in a gap between what was and what could have been (*my world is shrinking*). Although I understand that my life as a woman underpins POP (*a woman’s life takes its toll*), at the same time, POP has stolen my sense of being a women (*it has taken the woman out of me*). I see no beauty in me, and I have no sexual desire. Central to the experience of POP is a sense that I am living in silence: I am ashamed to talk about it (*prolapse is taboo*), I do not understand it (*what is going on down there?)* or what best to do about it (*is there anything that will make me better?*), and the health professional does not take me or my prolapse seriously or know what they are doing (*I am powerless in healthcare*). It would be a relief to talk about it so that I can get help, and to know that I am not alone (*it was a relief to tell someone*).

## Discussion

Our findings provide a conceptual synthesis, drawn from 37 international studies of 777 women, that can help us to understand the experience of women living with POP. We found that the physical losses of POP were intricately linked to loss of identity. We also found that women conceptualized POP as part of womanhood, yet also its thief. Finally, we found a vicious cycle of taboo, silence and misunderstanding about POP and its treatment. This silence was exacerbated by a sense that POP was not taken seriously in healthcare. Although we identified studies that explored healthcare professionals’ experience of treating overactive bladder [50], we did not identify any that explored the experience of treating women with POP. This area for future research may help us to understand some of the barriers to appropriate care.

There are some methodological issues related to QES to be considered: First, we identified 86% of included studies in Medline (76% from thesaurus terms). Although this might differ for each study, this finding resonates with our previous QES and indicates that Medline is an effective place to start searching. Further research on optimizing search strategies for QES would be useful as these are very time-consuming. Second, we would argue that verification of title and abstract screening by a second reviewer is not essential when the first reviewer is familiar with qualitative research methods: time might be more effectively spent on reflexive and collaborative analysis. QES searches do not aim to be exhaustive and there is no agreed guidance on whether a second reviewer is needed to verify screening. We would argue that verification of screening is not necessarily a good use of research time. Qualitative analysis does not involve numbers and does not rely on statistically representative data sets. Rather, the focus is on abstraction of ideas [8]. Third, we have not found that methodological appraisal, beyond *satisfactory* or *not satisfactory*, adds value to meta-ethnography. Similarly, we found it more useful to use a dichotomous categorisation (relevant or not relevant) rather than using all categories of relevance in GRADE-CERQual (partially, indirectly or unclear relevance). Finally, qualitative researchers should consider how, or whether *adequacy* (‘degree of richness and quantity of data’) and coherence (consistency across studies) are operationalizable for conceptual QES such as meta-ethnography. Our concern here is the misguided inference that *more* means *truer* [51]: ideas do not work like numbers. This concern resonates with issues related to sample size and data saturation in qualitative research [52]. However, we did find that keeping a tally of studies that supported each theme encouraged us to be reflexive and to challenge our interpretations. It also allows readers to identify studies that might be of interest to them.

Our findings are the result of a rigorous research process that incorporates the voices of 777 women living with POP. Our QES includes a diverse group of women from around the world and highlights shared experiences. However, we only included studies written in English, and further research focusing on experience of women from different backgrounds and contexts might be useful. People do not tend to define their experiences by pathology or condition, and it is not always possible to decipher isolated experiences. For this reason, we included eight studies of women with POP and urinary incontinence [15, 16, 25, 26, 31, 32, 37, 34]. We also found that urinary incontinence was an integral part of the experience of POP for some women in studies focusing primarily on POP.

Our conceptual model indicates that taboo and silence can become a barrier to healthcare. Findings resonate with a recent meta-ethnography of urinary incontinence [8]: a strong sense of stigma, shame and guilt, the pull towards concealment and secrecy, and the relief of being able to talk to others. The concept of taboo is relevant in healthcare and is associated with powerful emotions [53]. It is a poignant finding that women feel shame and disgust of their own bodies. In the seminal anthropological text, Purity and Danger, Douglas suggested that feelings of disgust stem from a cultural reaction to ambiguous categorisation or *liminality* [53]. This experience resonates with findings in urinary incontinence, where the ‘boundary between inside and outside the body is unconventionally and unexpectedly breached’ [8]. Our findings show that reducing shame and breaking silence can bring health and social rewards.

QES findings are a synthesis of primary research findings and, as such, are interpretations of interpretations (or third order constructs) [54]. This may mean that it is possible to lose sight of subtle nuances in the primary studies. Although qualitative research is traditionally *idiographic*, meaning that its focus is on unique contextual experience, we also feel that qualitative findings can contribute beyond a specific context. This meta-ethnography helps us to understand the global experience of living with POP. Our model illustrates the complex process of deciding what to do if you have a POP. Further studies to explore the complexity of decision making for POP within women’s health and urogynaecology services are timely, particularly in the context of the recent public concern regarding specific surgical procedures, which highlight the importance of incorporating patient voices into healthcare, policy and practice [3].
